# Using implementation research to understand lessons in reducing child mortality

**DOI:** 10.1186/s12887-023-04472-6

**Published:** 2024-02-28

**Authors:** Agnes Binagwaho, Lisa R. Hirschhorn

**Affiliations:** 1 University of Global Health Equity, Kigali, Rwanda; 2 Northwestern University Feinberg School of Medicine, Chicago, IL USA

**Keywords:** Implementation science, Low- and middle-income countries, Under-5 mortality, Implementation strategies, Amenable mortality, Contextual factors

## Abstract

Under-5 mortality decreased significantly worldwide between 2000 and 2015, but there is still progress to be made, particularly in lower- and middle-income countries. This supplement shares the work over the last four years on a project to understand how six countries (Bangladesh, Ethiopia, Nepal, Peru, Rwanda, and Senegal) were more successful in decreasing child mortality than many of their regional and economic peers. The use of implementation research across these countries identifies common implementation strategies and contextual factors that can facilitate or impede successful implementation of an evidence-based intervention and explores a common pathway to implementation. The work highlights how the use of implementation research to understand the “how” and the “why” behind countries’ success provides important actionable knowledge and lessons to country-level decision-makers, donors, and implementers as we arrive at the midpoint of the Sustainable Development Goal era.

## Using implementation research to understand lessons in reducing child mortality

Under-5 mortality (U5M) decreased significantly worldwide between 2000 and 2015, but there is still progress to be made, particularly in lower- and middle-income countries (LMICs). In 2020, the U5M rate in low-income countries was 13 times higher than in high-income countries [1], with higher rates among many rural areas and poorer communities. A significant number of these deaths result from amenable causes – preventable by a quality and responsive health system – including through evidence-based health system interventions known to reduce amenable mortality. These known interventions include vaccinations, quality care for malaria, diarrhea, pneumonia, and quality maternal care. The varied quality and application of these interventions reflect ongoing inequities in care delivery and access [2]. This disparity is a call to action: we have the interventions, but they are not being applied effectively and equitably across the globe. While there is robust literature on the effectiveness and coverage of these interventions, there is less work on capturing important insights into *what* was done (implementation strategies) and *what worked and why*, including barriers and facilitators which may have influenced strategy choice and success (contextual factors).

Implementation research provides the tools to deepen our understanding of successes and challenges in implementing existing and new evidence-based interventions (EBIs) globally, and is increasingly used in LMICs [3–6]. Understanding countries’ successes in accelerating the uptake and integration of existing and new EBIs into health systems, and the strategies used to do so, is key to closing the distance between research, policy, and practice.

This supplement shares the work over the last four years, funded by Gates Ventures and the Bill & Melinda Gates Foundation, on a project to understand how six countries (Bangladesh, Ethiopia, Nepal, Peru, Rwanda, and Senegal) were more successful in decreasing child mortality than many of their regional and economic peers. These countries were chosen based on review of existing data, geographic diversity, and input from an expert advisory group. The full case studies and description of the methodology are available at https://www.exemplars.health/topics/under-five-mortality and by Hirschhorn and colleagues [7]. Using mixed methods implementation research and case study methodology, the research teams used a shared framework [7] to extract country-specific, actionable knowledge on implementation strategies, context, and outcomes, focusing on amenable U5M. In addition, they explored the countries’ common and unique experiences in prioritizing contextual factors, implementation strategies, and implementation outcomes, across the five stages of implementation of EBIs: Exploration, Preparation, Implementation, Adaptation, and Sustainment (adapted from Aarons et al.) [8].

In this supplement, Sayinzoga and colleagues [9] investigate the contextual factors and strategies associated with Rwanda’s rapid uptake of two essential EBIs developed or adapted during the study period: rotavirus vaccine and effective prevention of mother-to-child transmission of HIV. In Nepal, Subedi and colleagues [10] describe how community health workers played an important role in delivery and acceptance of community-level EBIs, supported by other strategies which addressed barriers in the health care system, and discuss the importance of other non-health system factors. From Bangladesh, Huda and colleagues [11] found that implementation of facility-based integrated management of childhood illness and insecticide treated bed nets was successfully supported through the use of strategies including data use for decision-making, donor and implementing partner collaboration, small-scale testing, and an equity focus. In Ethiopia, Drown and colleagues [12] explore how the country leveraged its existing Health Extension Program as a platform for service delivery, allowing it to successfully introduce existing and new U5M-targeted EBIs nationally, improving feasibility and reach and supporting scale-up of these important interventions. In Peru, Garcia and colleagues [13] identify important contextual factors such as sustained economic growth and strong national prioritization of health initiatives. They identify implementation strategies including a focus on equity and utilizing research and data to guide health care decision-making and implementation as essential contributors to the country’s achievement of a remarkable reduction in U5M.

The supplement also shows the value of multiple-case study methodology to understand more generalized knowledge from these six countries including where challenges remain in equity. Binagwaho and colleagues [14] found that the six countries used a common pathway to implementation with a number of strategies common across EBIs and countries including use of data by decision-makers to identify problems and prioritize EBIs, determine implementation strategies and their adaptation, and measure outcomes. They identify common facilitators including culture of donor and partner coordination and culture and capacity of data use, and common barriers including geography and culture and beliefs. Ntawukuriryayo and colleagues [15], using facility-based delivery, focus on the important equity lens of implementation research [16, 17] to better understand the success and challenges in reducing inequity by wealth and by geographic region in each of the countries.

Reflecting on the approaches used by the overall project, in their commentary [18], Fernandes and colleagues describe the opportunities for an ecosystem where embedded research in programming is the new normal and how this will require changing and building incentives. Peterson [19] builds on the approaches used and proposes opportunities for more work in LMICs targeting the use of harmonized methodology for complex evaluations across multiple countries, which provides systematic results that are potentially more generalizable to accelerate U5M reductions in high-need settings.

## Conclusion

The use of implementation research across these countries identifies common implementation strategies and contextual factors that can facilitate or impede successful implementation of an EBI and explores a common pathway to implementation. The work highlights how the use of implementation research to understand the “how” and the “why” behind countries’ success provides important actionable knowledge and lessons to country-level decision-makers, donors, and implementers as we arrive at the midpoint of the Sustainable Development Goal era. As countries and the global community continue to work to reduce child, and particularly neonatal, mortality, we believe the supplement adds to the knowledge base in methods and strategies to accelerate the work to learn from the successes and adapt them to strengthen work needed in other countries.

## Data Availability

Not applicable (no data were generated during the study).

## Abbreviations

EBI: Evidence-based interventions
LMICs: Low-and middle-income countries
U5M: Under-5 mortality
